# A Service Evaluation to Evaluate the Predictors of Sexual Disinhibition on an Acute Male Inpatient Ward

**DOI:** 10.1192/bjo.2025.10531

**Published:** 2025-06-20

**Authors:** Robyn Wilcha, Manushini Angammana

**Affiliations:** South West London and St George’s Mental Health NHS Trust, London, United Kingdom

## Abstract

**Aims:** Sexual disinhibition, a neuropsychiatric symptom characterised by inappropriate sexual comments and/or behaviours, remains poorly understood in a general adult population. The absence of standardised assessment tools and limited measures to capture disinhibition may contribute to underestimating its prevalence and clinical significance. This study aimed to (1) determine the prevalence of sexual disinhibition on an acute all-male adult inpatient ward and (2) identify potential predictors of its occurrence.

**Methods:** Data from 55 patients was collected prospectively over a six-month duration to evaluate the prevalence and predictors of sexual disinhibition on an acute male inpatient ward. Included diagnoses encompassed psychosis, bipolar disorder, anxiety, depression, autism spectrum disorder, schizophrenia, schizoaffective disorder and personality disorders. Dichotomous variables included sexual disinhibition (current and past), delusions of a sexual nature, substance misuse, forensic history, history of abuse and medication use, including benzodiazepines, antipsychotics, mood stabilisers, antidepressants and depot medication. Scale variables included age. Family history of mental illness, age of onset, disease duration and unilateral parenting were excluded as a result of missing data. A binomial logistic regression was performed to examine the effects of these factors on the likelihood of sexual disinhibition.

**Results:** In total, 55 male patients, of whom 45.5% presented with sexual disinhibition, were included in our service evaluation (age: 44±14 years, detained under the MHA: 96.3%, previous sexual disinhibition: 61.8%, delusions of a sexual nature: 25.5%). Psychiatric diagnoses included psychosis (69.1%), bipolar disorder (16.4%), anxiety (18.2%), depression (27.3%), autism spectrum disorder (14.5%), schizophrenia (40.0%), schizoaffective disorder (21.8%) and personality disorder (25.5%). Presence of substance misuse was observed in 52.7% of patients, whilst forensic history was seen in 63.6%. Abuse was reported in nearly half of the patients (49.1%). The model was statistically significant (χ2(20)=45.329, P<0.001), explaining 75.1% (Nagelkerke R2) of the variance in sexual disinhibition and correctly classified 87.3% of cases. Only one variable was significant, delusions of a sexual nature (χ2(1) = 4.228, P=0.040).

**Conclusion:** Our findings highlight a positive association between sexual disinhibition and delusions of a sexual nature. Clinicians should recognise sexual disinhibition as a potential indicator of sexual delusions, aiming to assess these symptoms comprehensively and non-judgementally to better understand individual patient psychopathology. Educating patients and caregivers on this association may reduce stigma and aid understanding towards the patient. Future research should investigate the mechanisms of this relationship with larger sample sizes to minimise the risk of type II errors.

